# Exploring Protein–Protein Interaction in the Study of Hormone-Dependent Cancers

**DOI:** 10.3390/ijms19103173

**Published:** 2018-10-15

**Authors:** Yasuhiro Miki, Erina Iwabuchi, Katsuhiko Ono, Hironobu Sasano, Kiyoshi Ito

**Affiliations:** 1 Department of Disaster Obstetrics and Gynecology, International Research Institute of Disaster Science (IRIDeS), Tohoku University, Sendai 980-8575, Japan; kito@med.tohoku.ac.jp; 2 Department of Pathology, Tohoku University Graduate School of Medicine, Sendai 980-8575, Japan; e-iwabuchi@med.tohoku.ac.jp (E.I.); k-ono@patholo2.med.tohoku.ac.jp (K.O.); hsasano@patholo2.med.tohoku.ac.jp (H.S.)

**Keywords:** protein–protein interaction, co-immunoprecipitation, bioluminescence resonance energy transfer/förster resonance energy transfer, immunohistochemistry, in situ proximity ligation assay, super-resolution microscopy, estrogen receptor

## Abstract

Estrogen receptors promote target gene transcription when they form a dimer, in which two identical (homodimer) or different (heterodimer) proteins are bound to each other. In hormone-dependent cancers, hormone receptor dimerization plays pivotal roles, not only in the pathogenesis or development of the tumors, but also in the development of therapeutic resistance. Protein–protein interactions (PPIs), including dimerization and complex formation, have been also well-known to be required for proteins to exert their functions. The methods which could detect PPIs are genetic engineering (i.e., resonance energy transfer) and/or antibody technology (i.e., co-immunoprecipitation) using cultured cells. In addition, visualization of the target proteins in tissues can be performed using antigen–antibody reactions, as in immunohistochemistry. Furthermore, development of microscopic techniques (i.e., electron microscopy and confocal laser microscopy) has made it possible to visualize intracellular and/or intranuclear organelles. We have recently reported the visualization of estrogen receptor dimers in breast cancer tissues by using the in situ proximity ligation assay (PLA). PLA was developed along the lines of antibody technology development, and this assay has made it possible to visualize PPIs in archival tissue specimens. Localization of PPI in organelles has also become possible using super-resolution microscopes exceeding the resolution limit of conventional microscopes. Therefore, in this review, we summarize the methodologies used for studying PPIs in both cells and tissues, and review the recently reported studies on PPIs of hormones.

## 1. Introduction

The quaternary structure of proteins, such as their dimerization, is achieved via specific and non-specific non-covalent interactions. Non-specific interactions occur due to hydrophobic effects or van der Waals forces, and specific interactions occur due to electrostatic forces. It is well known that hydrophobicity is the main driving force behind protein folding [1]. Electrostatic interactions are also known to play pivotal roles in protein folding, stability, flexibility, and function [1]. Protein–protein interaction (PPI) has also been demonstrated to be important in determining protein function. For instance, epidermal growth factor receptor (EGFR) binds adaptor proteins, such as growth factor receptor binding protein 2 (Grb2), via an intracellular phosphorylation site in order to activate the downstream signaling cascade [2,3]. In addition to growth signaling, Grb2 is required for endocytosis of EGFR, which is mediated by clathrin [4]. Prior to the activation of intracellular signaling, EGFR binds to its ligands and undergoes homodimerization or heterodimerization with another EGFR family receptor, such as the human epidermal growth factor receptor (HER) 2, HER3, and HER4 [5].

**Figure 1:**
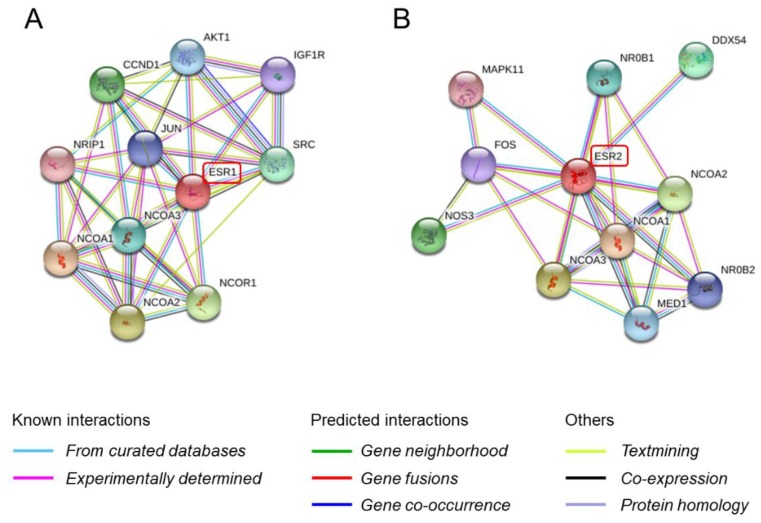
Prediction using the STRING database (https://string-db.org/). (**A**) ESR1 protein (ERα) interaction on confidence prediction. AKT1, V-akt murine thymoma viral oncogene homolog 1; CCND1, Cyclin D1; IGF1R, Insulin-like growth factor 1 receptor; NRIP1, Nuclear receptor interacting protein 1; JUN, Jun proto-oncogene; SRC, V-src sarcoma (Schmidt-Ruppin A-2) viral oncogene homolog (avian); NCOA, Nuclear receptor coactivator; NCOR1, Nuclear receptor corepressor 1. (**B**) ESR2 protein (ERβ) interaction on confidence prediction. MAPK11, Mitogen-activated protein kinase 11; NR0B1, Nuclear receptor subfamily 0, group B, member 1; DDX54, DEAD (Asp-Glu-Ala-Asp) box polypeptide 54; FOS, FBJ murine osteosarcoma viral oncogene homolog; NOS3, Nitric oxide synthase 3 (endothelial cell); NCOA, Nuclear receptor coactivator; MED1, Mediator complex subunit 1; NR0B2, Nuclear receptor subfamily 0, group B, member 2.

In the nuclear receptor super family, dimerization (homodimerization or heterodimerization) is a crucial process for the activation of nuclear receptors after ligand binding. Activated nuclear receptors then recruit cofactors forming a receptor–cofactor complex. Among all the nuclear receptor super families, the estrogen receptor (ER) is most well studied because it plays an important role in hormone-related growth in hormone-dependent cancers, especially breast and endometrial cancers [6,7]. ER has several functional domains which include the ligand binding domain (LBD), DNA binding domain (DBD), hinge region, and two activation function domains (AF-1 and AF-2) [8,9,10]. Ligands such as estradiol induce ER dimerization and its subsequent binding to estrogen response elements located in the promoter regions of ER-target genes, upregulating their transcription [11]. Two isoforms of ER, ERα (NR3A1) and ERβ (NR3A2) have been well characterized so far. In addition, it is well known that ERβ has five splicing variants, ERβ1-5 (ERβ2 is also known as ERβcx) [12,13]. ERα (66 kD) is also reported to have several splicing variants, two of which are ERα-36 (kD) and ERα-46 (kD) [14,15]. Binding of the ligands to the ER usually leads to ER dimerization, forming ERα homodimers, ERβ homodimers, and ERα/ERβ heterodimers in the nucleus [16,17]. Although the pattern of formation of heterodimers with ER variants remains unclear, it is suggested that the heterodimers may regulate a different set of genes than the homodimers. Recruitment of the dimer, along with co-regulators, known as nuclear receptor co-repressors (NCORs) and nuclear receptor *co*-activators (NCOAs), is required to exert transcriptional activity of ER dimers on target genes [18]. The PPIs between ER and its coregulators can be predicted using the in silico Search Tool for Retrieval of Interacting Genes and proteins (STRING) [19] (Figure 1).

Tamoxifen, which is a selective ER modulator and has an inhibitory effect on estrogen-dependent transcription in breast cancer, inhibits recruitment of NCOA1-3 and induces recruitment of NCOR1 and NCOR2 [18,20,21,22].

Results of those described above indicated that evaluation of intracellular PPIs could offer significant advantages for understanding hormonal signaling in hormone-dependent cancers. Many methodologies have been proposed for the evaluation of PPI [23]. PPI analysis by in vivo imaging using xenograft or transgenic mice is possible [23,24]. Although gene recombination and antibody engineering technologies are employed for investigation of PPI, it is mostly examined using in vitro cell culture models. In addition, using appropriate technologies for PPI analysis, the dynamics of intracellular PPIs in living cells could be visualized at in vitro levels [25,26,27]. Novel technologies of in vitro PPI examination have been introduced [28,29,30]. On the contrary, detection of intracellular PPI by histological and pathological analyses using tissue samples is considered difficult, as compared to in vitro analysis using cultured cells.

## 2. Co-Immunoprecipitation

Co-immunoprecipitation (Co-IP) has been classically used for detection of PPI in vitro. Co-IP includes using an antigen–antibody complex as a bait and the target interacting protein as prey. Typically, cell lysate is reacted with the bait-specific antibody. Along with the bait protein and any bait-associated proteins, the prey protein (the interaction partner) will be co-precipitated. The bait protein interacts with its specific antibody, which is bound to micro-beads, such as agarose, sepharose, or magnetic beads [31]. Under the presence of the prey protein that binds to bait protein in the sample, a bait–prey complex will be formed and it will be co-precipitated. Subsequently, the prey protein can be detected by downstream analysis, such as the western blot. If the prey protein is unknown, advanced proteomic analysis such as mass spectrometric analysis can be employed for its identification [32]. Co-IP does not prove that the two target proteins are directly bound to each other, because the presence of a third protein cannot be ruled out.

In hormone-dependent cancer research, it is well known that ERα is co-immunoprecipitated with NCOR in MCF-7 cells treated with tamoxifen, but not in untreated or estradiol-treated cells [21]. Mohammed H et al. [33] reported the presence of estrogen-enriched ER interactive proteins by using a novel technique called rapid immunoprecipitation mass spectrometry of endogenous proteins (RIME). Among 108 ER-associated proteins extracted, GREB1 was the most significantly increased as an estrogen-ER-interactive protein [33]. GREB1 is demonstrated to have an essential role in ER-mediated transcription. The principle of the co-IP itself does not vary; however, downstream analysis of the new target proteins affecting hormone signal are being constantly developed in hormone-dependent cancers.

## 3. Bioluminescence Resonance Energy Transfer and Förster Resonance Energy Transfer

Resonance energy transfer (RET) technologies such as bioluminescence RET (BRET) and Förster (or Fluorescence) RET (FRET) is also used widely to examine PPI in vitro [34,35]. FRET uses the principle that an acceptor molecule (fluorophore) absorbs energy emitted from an excited donor molecule (fluorophore). Detection of FRET is usually done by irradiating the donor using its excitation wavelength and measuring the fluorescence intensity of the donor or acceptor [36,37]. Cyan variant of GFP (CFP) and yellow version of GFP (YFP) are usually employed as donor and acceptor molecules, respectively [37,38]. During detection of the donor’s fluorescence intensity, if the donor and acceptor are near, the energy derived from the donor is absorbed by the acceptor, thus the fluorescence intensity of the donor decreases. When both molecules are relatively separated, the fluorescence intensity of the donor increases. When measuring the fluorescence intensity corresponding to the acceptor, the results are reversed. Likewise, the principle of BRET is based on energy transfer between fusion proteins containing Renilla luciferase (RLuc) from Renilla reniformis as a donor molecule, and fluorophores such as GFP and YEP as acceptor molecules [39,40]. Since bioluminescence technology is used in BRET, it has an advantage over FRET because it does not require an excitation light source and its corresponding system for the donor [34]. Therefore, BRET is applicable to various PPI examinations including mouse in vivo imaging [23,24,41,42]. BRET can be used for studying deep tissues of mouse as it uses a highly sensitive cooled charge coupled device (CCD) camera or complementary metal oxide semiconductor (CMOS)-based optical imaging instrument for detection [42,43,44].

ER dimerization was reported using both FRET [17] and BRET [45]. Michelini et al. [45] established a monitoring system for estrogen-like compounds using BRET of ERα homodimerization as an index of ERα activation. ERα proteins fused with Rluc (donor) and enhanced YFP (acceptor) were used for BRET in their study [45]. Techniques such as BRET have been used for PPI analysis. The AlphaScreen assay from PerkinElmer (Waltham, MA, USA) uses as a photosensitizer (phthalocyanine), which converts ambient oxygen (O_2_) present in the donor beads to singlet oxygen (^1^O_2_) through excitation [46]. When acceptor beads are near the donor, i.e., the distance is less than 200 nm, energy is transferred from ^1^O_2_ to the thioxene in the acceptor, and radiation (520 to 620 nm) is emitted from the acceptor. Interaction of ER and retinoic acid receptor with their co-activator was screened using the AlphaScreen assay [47]. These findings suggest that RET technologies, including AlphaScreen, are useful methods for screening possible hormone–receptor interactions and interactions with coregulators.

## 4. Protein Detection Methods

Several protein detection methods are available for in vitro, ex vivo, and in vivo analysis. In 1960, radio immunoassay (RIA) was established for measurement of insulin in plasma by Yalow, a Nobel Prize laureate [48]. Enzyme immunoassay (EIA) and enzyme-linked immunosorbent assay (ELISA) are non-radioisotope methods developed and used traditionally to quantitatively detect target proteins or antibodies in various type of samples such as serum, plasma, cell lysate, and tissue homogenate [49,50,51]. Western blotting, which includes gel electrophoresis of the protein, followed by electrophoretic transfer to a membrane, is also a well-established method [52,53]. Western blotting has the advantage that it can be used to determine the molecular weight of target proteins, while RIA, EIA, and ELISA can be used for quantification of the molecules. In addition to the analysis described above, multiplex analyses are also becoming a popular technique for the detection of target proteins and their interaction partners. Antibody array enables detection of many target proteins, including cytokines and phosphorylated proteins [54,55]. Flow cytometric analysis could also be used to detect protein expression in cells using antigen–antibody reactions. Furthermore, the technique of simultaneously detecting multiple cytokines by combining bead-based labeling technology and flow cytometry (multiplex cytokine assay) has been frequently used [56]. However, the protein detection methods described above cannot distinguish the intracellular or intratissue localization of target proteins.

**Figure 2:**
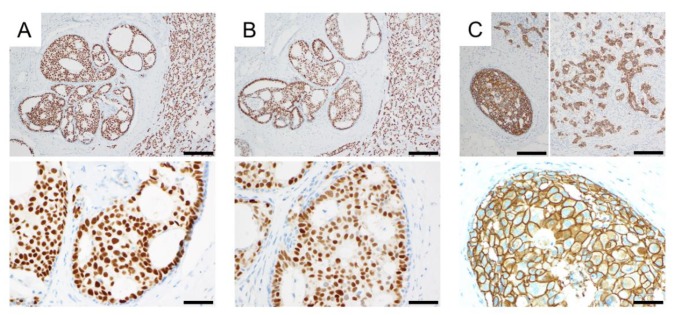
Immunohistochemistry of biomarker proteins used for pathological diagnosis of breast cancer subtypes. Immunostaining for ERα (**A**); progesterone receptor (**B**); and HER2 (**C**) was performed using autostainer, Ventana Benchmark ULUTRA staining system (Roche). Immunoreactivities of ERα and progesterone receptor were detected in the nucleus of breast carcinoma cells. HER2 was detected in cell membranes of breast carcinoma cells. Top photographs are of a lower magnification (scale bar, 200 μm), and bottom photographs are of a higher magnification (scale bar, 50 μm).

Immunohistochemistry is a classical microscopy-based technique for visualizing the target protein in tissues and cells using a specific antibody. Immunohistochemistry was invented in 1941 by Coons and co-investigators [57] and is a principal technique for pathological diagnosis. For example, in hormone-dependent cancers, ER, progesterone receptor (PgR), HER2 and Ki67 are routinely examined by immunohistochemistry (Figure 2) for planning therapeutic strategies for breast cancer patients. According to evaluation based on these biomarkers, breast cancer is divided into four subtypes namely, luminal A, luminal B, HER2 type, and triple-negative type mostly based upon results of immunohistochemistry above. Using the companion diagnostic assay, which detects a predictive biomarker, patients with cancer were classified as responders and non-responders to its target therapy. HER2 test using immunohistochemistry, which is named “HercepTest (Dako, Carpinteria, CA, USA) [58]”, is considered as the prototypic companion diagnostic employed in immunohistochemistry [59].

**Figure 3:**
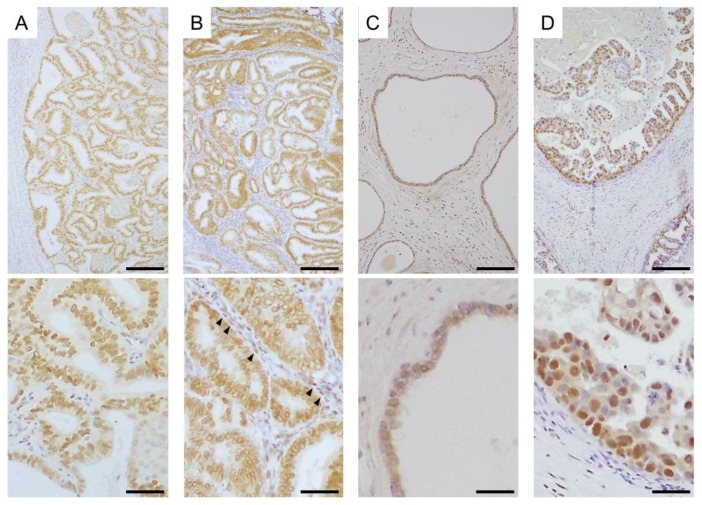
Immunohistochemistry of steroid hormone receptors in hormone-dependent cancers. (**A**) Expression of ERα in endometrial carcinoma tissue; (**B**) Expression of androgen receptor (AR) in endometrial carcinoma tissue. AR was detected in both carcinoma cells and stromal cells (arrow heads) in this case; (**C**) Expression of ERβ in non-pathological epithelia of prostate tissue; (**D**) Expression of ERβ5 in breast carcinoma tissue. Top photographs are of a lower magnification (scale bar, 200 μm), and bottom photographs are of a higher magnification (scale bar, 100 μm).

Immunohistochemistry is currently widely used at the global level. It is possible to identify the localization of a target protein in normal tissues and disease in routinely processed tissue specimens by immunohistochemistry. Steroid hormone receptors can also be detected by immunohistochemistry in several hormone-dependent cancers (Figure 3). Immunocytochemistry can be used for examining the subcellular localization of proteins in single cells.

**Figure 4:**
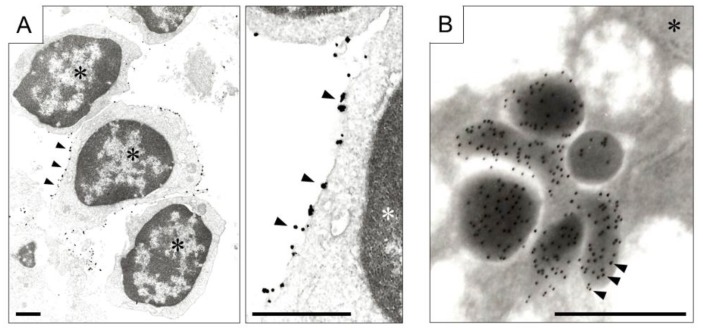
Immunoelectron microscopy. (**A**) Expression of chemokine receptor (CXCR, arrow heads) on the membrane of lymphocytes aggregated in colitis. Left photograph is at low magnification, and right is at high magnification (scale bar, 1 μm). Gold colloid conjugated antibody for CXCR, and the signal was amplified by the silver nanoparticles. Asterisks indicate nuclei of lymphocytes; (**B**) Secretion of 5-hydroxytryptamine (arrow heads) from gastric cancer cells. Colloidal Gold Conjugated secondary antibody was employed (scale bar, 0.5 μm). The asterisk indicates nucleus of gastric carcinoma cell.

In addition, protein localization at the organelle level can be detected using transmission and immunoelectron microscopes (Figure 4). In immunoelectron microscopic analysis, secondary antibodies labeled with gold or silver colloids are allowed to interact with primary antibodies and target protein complex. Immunoelectron microscopy is traditionally known to be effective in detecting microbial pathogens [60]. In addition, in renal diseases such as monoclonal gammopathy, immunoelectron microscopic analysis provides useful information for diagnosis [61,62]. In cultured cell as well as tissue diagnosis, immunoelectron microscopic analysis provides significant intracellular information, which cannot be obtained using a light microscope [63].

Kocanova et al. [64] demonstrated that the intranuclear accumulation pattern of ERα in SK19 varied depending on estradiol, SERM and ER-inhibitor (ICI182,780) by using confocal laser scanning microscopy. In addition, the accumulation pattern of ERα observed by confocal microscopy was also confirmed in MCF-7 cells by immunoelectron microscopic analysis [64]. ERα molecules labeled with gold particles were detected within 100 nm from each other in estradiol or ICI182,780 treated MCF-7 cells [64]. This study also made it possible to visualize the intranuclear localization of ERα and 20S proteasome subunit α2 (α2) in SK12 cells by double-immunonanogold labeling approach [64]. ERα was labeled with 10 nm gold particles and α2 was labeled with 6 nm gold particles, respectively. In estradiol treated MCF-7 cells, interaction of ERα and α2 in the nuclear microdomains of about 100 nm in diameter was observed [64].

## 5. Proximity Ligation Assay

**Figure 5:**
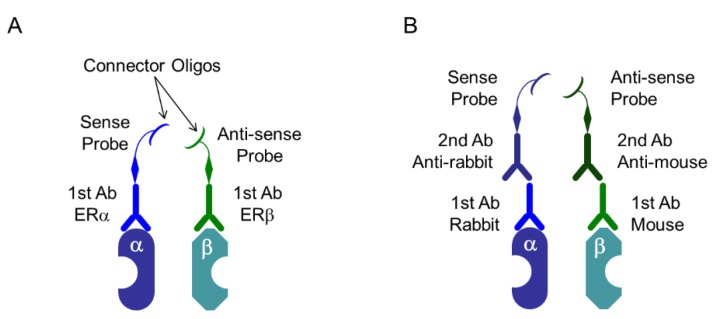
Proximity ligation assay (PLA) system: specific probes. (**A**) Specific primary antibodies (1st Ab) are linked to special oligonucleotide probes, that are sense and antisense oligos named as PLUS oligo and MINUS oligo, respectively. In this case, primary antibodies derived from both, same and different species can be used; (**B**) PLUS oligo and MINUS oligo link to secondary antibodies (2nd Ab) derived from mouse, rabbit, or goat, and it depends on the species of the primary antibodies. In this case, primary antibodies derived from different species should be used. In this Scheme, as an example, heterodimer of ERα and ERβ are depicted.

**Figure 6:**
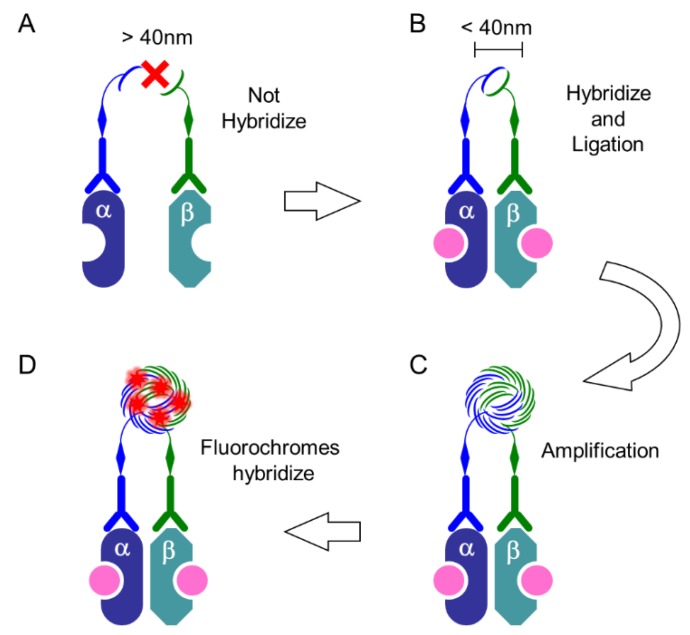
PLA system: procedure. (**A**) When the distance between two targets is more than 40 nm, PLUS oligo and MINUS oligo, which is linked to primary or secondary antibodies, cannot hybridize with each other; (**B**) When these probes are present at less than 40 nm, they hybridize and then ligate to form a circle; (**C**) The DNA circle undergoes several hundredfold replication by the rolling-circle amplification (RCA) reaction; (**D**) The fluorescently labeled oligonucleotides will hybridize to the RCA product. In this Scheme, as an example, heterodimer of ERα and ERβ induced by estrogen-binding (pink) are depicted.

Advanced techniques based on immunohistochemistry have been developed, and in situ proximity ligation assay (PLA) was developed to visualize PPI of two proteins in tissue samples [65,66]. This method has been used to detect PPIs and post-translational modifications (PTMs) by enabling target proteins to interact with specific antibodies, which ligate with special oligonucleotides, which are subsequently amplified. The principle of in situ PLA is summarized in Figure 5 and Figure 6. Dual targets of primary antibodies (e.g., one that recognizes ERα and another that recognizes ERβ) were employed to detect PPI. During in situ detection of PTM, two specific antibodies, such as antibodies against EGFR and the phosphorylated form of EGFR, are selected for in situ PLA. The PLA kit (Duolink PLA) sold by Olink Bioscience (Uppsala, Sweden), and now available from Sigma-Aldrich/Merck (Darmstadt, Germany) was used.

**Figure 7:**
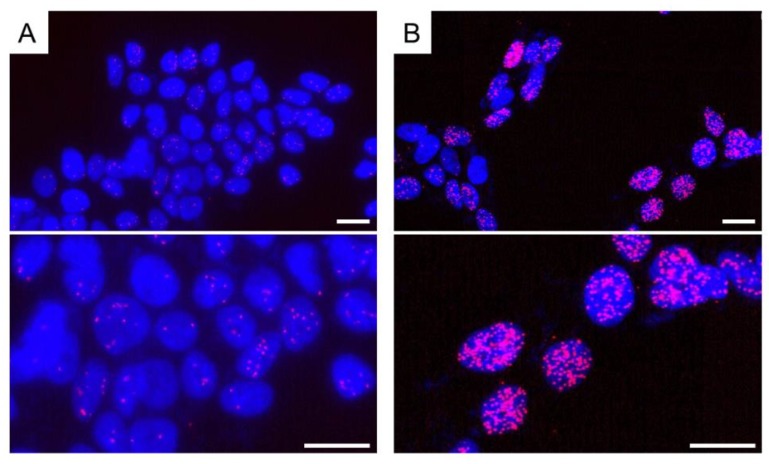
Detection of ERα homodimer in MCF-7 cells by in situ PLA. The cells were grown on cover slides. After fixation with 4% paraformaldehyde, cells were permeabilized using 0.1% Triton-X-100. (**A**) A few PLA signals (red dots) were detected in vehicle (dimethyl sulfoxide)-treated cells as a control. Antibodies for ERα were obtained from rabbit monoclonal antibody SP-1 (Abcam, Cambridge, UK) and mouse monoclonal antibody 6F11 (Leica Biosystems, Wetzlar, Germany); (**B**) When estradiol (10 nM) was used for treatment, PLA signals (red dots or clusters) significantly increased. Nuclei were stained blue (DAPI). PLA signals were red (Texas red). Top photographs are of a lower magnification, and bottom photographs are of a higher magnification. Scale bar, 10 μm.

**Figure 8:**
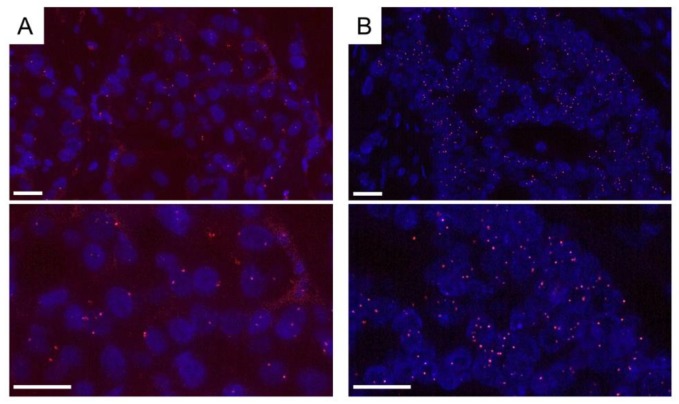
Detection of ERα homodimer in breast carcinoma tissue by in situ PLA. Formalin-fixed paraffin-embedded (FFPE) samples were used for pathological diagnosis. ERα antibodies, SP-1 and 6F11 were employed. Both cases with low- (**A**) and high-PLA score (**B**) were demonstrated. PLA signals (red dots) were detected in the nucleus of breast carcinoma cells. Nuclei were stained blue (DAPI). PLA signals were stained red (Texas red). Top photographs are of a lower magnification, and bottom photographs are of a higher magnification. Scale bar, 20 μm.

We recently demonstrated that ER dimerization was induced by estrogen treatment in breast cancer cell lines (Figure 7). ERα homodimers in MCF7 cells treated with estradiol for 15, 45, and 90 min were detected in the nucleus compared to their localization in the control cells [63]. In this study, we employed a scoring system for the PLA signal, which is quantified as the area of PLA-fluorescence dots in the nucleus using an image analyzer (Lumina Vision, Mitani Corp., Fukui, Japan). The PLA score of ERα homodimer was obtained by estradiol treatment after 15 to 45 min in MCF-7 cells. These findings did indicate that the interaction of estrogen with ER could be visualized by the detection of ERα homodimer by using in situ PLA. We also detected both ERα homodimer (Figure 8) and ERα/β heterodimer by PLA in breast carcinoma tissues, which were 10% formalin-fixed paraffin-embedded (FFPE) for pathological diagnosis [67]. It was impossible to detect ER activation in breast carcinoma tissues, although PLA technology might be able to resolve this issue in the future.

In addition, we also reported the visualization of heterodimers of ERβ variants in breast carcinoma cell lines [63,68]. In a previous study, the PLA score of ERα/β1 heterodimers increased after 15 min of stimulation with estradiol, though there was no increase in the PLA score of ERα/β2 or ERα/β5 heterodimers in MCF-7 cells stimulated by estradiol. It is reported that ERβ2 could form heterodimers with ERα or ERβ1 in a ligand independent manner [69,70]. Ligand independent dimerization of ERβ5 remains unknown, but the binding affinity of ERβ5 for estradiol is known to be lesser than that of ERβ1 [71]. In general, the isoforms of ERβ prevent ERα-dependent transcriptional activities, thus preventing the formation of the heterodimer (ERα/β). However, these inhibitory effects are thought to vary according to the isoforms [72]. In situ PLA analysis using normal and cancerous human tissues may clarify the significance of the heterodimer patterns observed in each ERβ and ERα isoform. Flanders et al. [73] demonstrated the presence of the Smad complex (Smad2/3-Smad1/5/9) in breast cancer tissues using tissue microarray and brightfield PLA. In brightfield PLA, horseradish peroxidase (HRP)-conjugated oligonucleotides were employed instead of fluorescence labeled oligonucleotides [74]. Intratumoral binding of estradiol and ER in breast cancer tissues was visualized by PLA using specific antibodies [74]. In the presence of specific antibodies, the hormone receptor-binding assay using tissues by PLA could be possible.

Interaction of androgen receptor with JunD in prostate cancer LNCaP cells was identified by PLA, and this interaction was inhibited by a selected lead chemical [75]. We recently reported the interaction of HER2 with carcinoembryonic antigen-related cell adhesion molecule 6 (CEACAM6) in breast cancer tissues [76]. We further confirmed that the PLA score of HER2/CEACAM6 was significantly associated with efficacy of HER2 inhibition after treatment with trastuzumab in breast cancer patients [76]. With respect to the PLA of HER2, a heterodimer with HER3 has been reported, and its effect in the inhibition of the HER2 homodimer formation has been investigated [77,78,79,80]. It was reported that Pan-HER and EGFR-targeting monoclonal antibody inhibited binding of EGFR to its receptor and disrupted EGFR-dimerization levels which were evaluated by PLA [81]. Moreover, DNA damage-induced protein complexes in acute lymphoblastic leukemia cell lines were examined using PLA [82]. The reports described above suggest the possibility of PLA as a diagnostic tool for prediction of efficacy and resistance towards target therapies in several types of cancer, including hormone-dependent cancers. Quantification of the amplified connector oligos by quantitative PCR, and quantification of fluorescence signals obtained through flow cytometry have been studied using PLA [82,83,84] thus it can be used as a diagnostic tool [85].

## 6. Super-Resolution Microscopy

**Figure 9:**
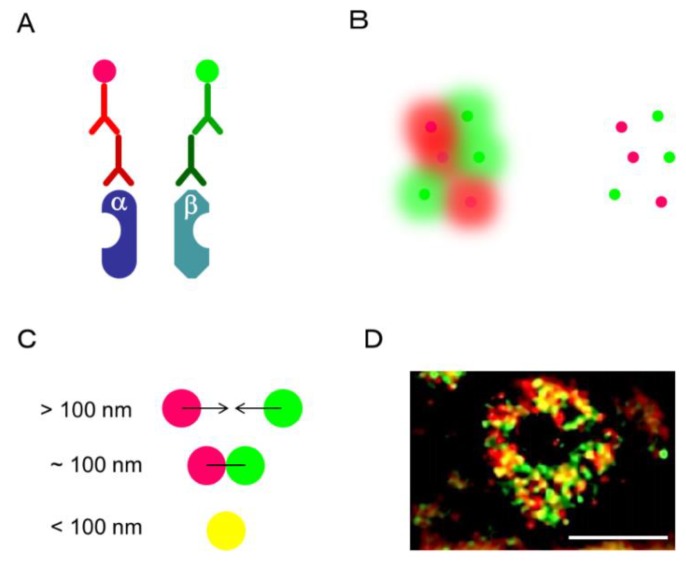
SIM analysis. In this case, ERα homodimer was detected. ERα antibodies, rabbit monoclonal SP-1 and mouse monoclonal 6F11 were employed. (**A**) Secondary antibodies were labeled with Alexa Fluor 594 (Red) and Alexa Fluor 488 (Green), respectively. (**B**) In conventional fluorescence microscopy (Left), the fluorescence signal from the entire molecule in the sample is simultaneously detected, and the fluorescence from each molecule overlaps. In SIM analysis, fluorescence from only a small part of the fluorescent molecule is detected, making it possible to determine the exact position of the molecule [93]. (**C**) When the two molecules are located at 100 nm or more, they can be recognized as red and green fluorescence, respectively. When two molecules are close to each other (<100 nm), they can be visualized with a marginal yellow fluorescence. (**D**) Intranuclear localization of ERα in MCF-7 was observed by N-structured illumination microscopy (SIM). Scale bar, 5 μm.

Optical microscopy has contributed immensely to the elucidation of the microstructure of cells, but the resolution of an optical microscope is limited because of its diffraction limit, explained by the Abbe’s law [86,87]. Intranuclear distributions of ER and AR have been examined with a confocal laser scanning microscope (LSCM) [88,89,90,91]. An intranuclear formation of both ER and AR dimers has been well known to change after their treatment with ligands. LSCM has been conducted to observe the intracellular localization of target proteins as it has a resolution of about 200 nm, which is recognized as the optical diffraction limit [86]. The Nobel Prize in Chemistry in 2014 was awarded for the discovery of super-resolved fluorescence microscopy, where resolution beyond the diffraction limit was achieved. Recently, several types of super-resolved fluorescence microscopes have been developed which have a high resolution, such as structured illumination microscopy (SIM), stimulated emission depletion (STED) microscopy, photoactivated localization microscopy (PALM), fluorescence photoactivation localization microscopy (fPALM), (direct) and the stochastic optical reconstruction microscopy (STORM/dSTORM) [81,82,87]. The resolutions of each of these super-resolution microscopes are as follows: SIM, 100 nm; STED, 30–70 nm; PALM/fPALM, 10–55 nm, and STROM/dSTROM, 10–55 nm [92]. Among these super-resolution microscopes, SIM is considered the most accessible. In Figure 9, immunofluorescence of ERα recognized by two different monoclonal antibodies in MCF-7 was captured by Nikon’s SIM (N-SIM, Nikon, Tokyo, Japan) with a lateral resolution of approximately 100 nm [68]. In this case, the secondary antibodies against the two primary antibodies are labeled in fluorescence red and green, respectively. Therefore, when the two antigens are near, i.e., within 100 nm, yellow fluorescence is observed. In MCF-7, red, green, and yellow fluorescence was detected in the nucleus. Red and green indicate ERα monomers, yellow is considered to include ERα homodimer. In our previous research, the intratumoral yellow area in MCF-7 significantly increased after treatment with estradiol [68]. A similar result was obtained by PLA analysis. Although SIM cannot be used to visualize a larger region, it has the advantage that the change in the number and distribution of the intraorganelle target signals, including those of the nucleus, can be evaluated.

Gonadotrophic hormones such as follicle-stimulating hormone (FSH) and luteinizing hormone (LH) derived from the pituitary gland, bind to their corresponding receptors in ovarian granulosa (FSH-R) and theca (LH-R) cells, respectively. Jones and *co*-investigators [94] demonstrated the existing patterns of LH-R, which were monomers, dimers, and various oligomers, in HEK293 transfected Tag-labeled LH-R using photoactivatable dye (PD)-PALM [94]. Dimerization pattern of LH-R and FSH-R was also revealed using PD-PALM in HEK293 transfected Tag-linked LH-R and FSH-R [95]. LH-R is demonstrated to interact with FSH-R in the form of distinct heterooligomers as well as simple heterodimers [95]. In breast cancer cell lines, cluster formation, including formation of homo and heterodimers of HER family (HERs) members, such as HER2 and HER3, was examined by spectral precision distance/position determination microscopy (SPDM) [96,97]. HERs cluster formation and the distance between the clusters were different in normal epithelia and cancerous cell lines; furthermore, they changed after treatment with the HER3 ligands and were sensitive to the HER2 inhibitor [96,97]. Therefore, visualization of the intracellular microenvironment by super-resolution microscopy is useful for elucidating the steroid function and predicting the drug efficacy based on the receptor dimer formation pattern.

In our previous study, immunoreactivity of CEACAM6 in breast carcinoma tissues was not associated with the efficacy of HER2 inhibitor, trastuzumab, in the breast cancer patients examined [76]. However, interaction of CEACAM6/HER2 visualized by in situ PLA was significantly associated with the efficacy of trastuzumab treatment [76]. Therefore, detection of PPI could better enable evaluation of intracellular signal transduction in cancer cells in pathological specimens. The assumed maximum distance between antigen recognition sites of target proteins is <40 nm (10–30 nm [98]) using probe-linked primary antibodies, and increases if probe-linked secondary antibodies are used [98]. Furthermore, PPI is well known to be complex, with mediator proteins required to detect the interaction between the two target proteins. The issue with regard to the detection limit of “distance” is important, as in any other method, and it will be difficult to prove whether the two proteins are directly or indirectly coupled with each other. Two or more PPI analysis methods are required to confirm an analysis [98]; thus, it may be useful to refer to a simulation or prediction analysis software, which is available online [19,99]. Antibody technology is extensively utilized in PPI detection methods, therefore confirmation of the specificity of the antibody to the antigen should be done carefully and reliably.

## 7. Future Perspectives on Exploring PPI in Hormone-Dependent Cancers

Inhibitors that directly target estrogen receptor/co-activator binding (co-activator binding inhibitors: CBI) have been developed [100,101,102]. Both ERα and ERβ bind co-factor proteins through LxxLL motifs. It is reported that tetrahydro-iso-alpha acid inhibits the estrogen-stimulated transcriptional activity of ERs by interfering with the binding of ERs to LxxLL motifs in breast cancer cell line MCF-7 [102,103]. One of the enzothiophenone derivatives also has an inhibitory action on the binding of ERα to LxxLL, and the compound also represses the transcription of the mutant form of ERα gene (ESR1) [104]. Hormone- or endocrine-therapy has been applied to patients with ERα-positive breast cancer, and it has been noted that ESR1 mutation is involved in endocrine therapy resistance [105]. Therefore, CBI is considered beneficial for endocrine therapy resistant cases too.

Since scanning tunneling microscopy and atomic force microscopy (AFM) were developed in the 1980s, a wide variety of scanning probe microscopy (SPM) techniques have been developed based on their principles [106]. The principle of SPM is that a sharp-tipped probe closely scans the surface of the sample and an image is obtained by scanning the probe, while keeping the physical quantity exchanged between the probe and the sample constant [106,107]. Understanding biomaterials at the nanoscale has advanced by the development of STM technology. It is possible to detect ER dimers using SPM, and SPM is considered as a usefull tool for assessing compouds which have the ability to either active or inhibit ER dimerization [108, 109, 110]. Since estrothiazine, like natural estrogens, shows ER dimerization [108, 109, 110] as evaluated by AFM analysis, it is considered as an ER agonist [109, 110].

In recent years, PPI has been considered one of the important concepts in drug discovery, and PPI itself is regarded as a drug target. PPI is a key mechanism in understanding steroid hormone signals, and is an indispensable part of hormone-dependent cancer research. Furthermore, evaluation of PPI is thought to provide useful information, not only as a diagnostic tool for disease, but also as a predictive marker for the efficacy of the molecular-targeted agents.

## References

[1] Kumar S., Nussinov R. (2002). Close-range electrostatic interactions in proteins. ChemBioChem.

[2] Lowenstein E.J., Daly R.J., Batzer A.G., Li W., Margolis B., Lammers R., Ullrich A., Skolnik E.Y., Bar-Sagi D., Schlessinger J. (1992). The SH2 and SH3 domain-containing protein GRB2 links receptor tyrosine kinases to ras signaling. Cell.

[3] Rozakis-Adcock M., McGlade J., Mbamalu G., Pelicci G., Daly R., Li W., Batzer A., Thomas S., Brugge J., Pelicci P.G. (1992). Association of the Shc and Grb2/Sem5 SH2-containing proteins is implicated in activation of the Ras pathway by tyrosine kinases. Nature.

[4] Huang F., Khvorova A., Marshall W., Sorkin A. (2004). Analysis of clathrin-mediated endocytosis of epidermal growth factor receptor by RNA interference. J. Biol. Chem..

[5] Jorissen R.N., Walker F., Pouliot N., Garrett T.P., Ward C.W., Burgess A.W. (2003). Epidermal growth factor receptor: Mechanisms of activation and signalling. Exp. Cell Res..

[6] Sasano H., Harada N. (1998). Intratumoral aromatase in human breast, endometrial, and ovarian malignancies. Endocr. Rev..

[7] Ito K., Utsunomiya H., Yaegashi N., Sasano H. (2007). Biological roles of estrogen and progesterone in human endometrial carcinoma—New developments in potential endocrine therapy for endometrial cancer. Endocr. J..

[8] McInerney E.M., Katzenellenbogen B.S. (1996). Different regions in activation function-1 of the human estrogen receptor required for antiestrogen- and estradiol-dependent transcription activation. J. Biol. Chem..

[9] Delaunay F., Pettersson K., Tujague M., Gustafsson J.A. (2000). Functional differences between the amino-terminal domains of estrogen receptors α and β. Mol. Pharmacol..

[10] Metivier R., Penot G., Flouriot G., Pakdel F. (2001). Synergism between ERα transactivation function 1 (AF-1) and AF-2 mediated by steroid receptor coactivator protein-1: Requirement for the AF-1 α-helical core and for a direct interaction between the N- and C-terminal domains. Mol. Endocrinol..

[11] Beato M. (1989). Gene regulation by steroid hormones. Cell.

[12] Leygue E., Dotzlaw H., Watson P.H., Murphy L.C. (1999). Expression of estrogen receptor β1, β2, and β5 messenger RNAs in human breast tissue. Cancer Res..

[13] Cavallini A., Messa C., Pricci M., Caruso M.L., Barone M., Di Leo A. (2002). Distribution of estrogen receptor subtypes, expression of their variant forms, and clinicopathological characteristics of human colorectal cancer. Dig. Dis. Sci..

[14] Wang Z., Zhang X., Shen P., Loggie B.W., Chang Y., Deuel T.F. (2005). Identification, cloning, and expression of human estrogen receptor-α36, a novel variant of human estrogen receptor-α66. Biochem. Biophys. Res. Commun..

[15] Rao J., Jiang X., Wang Y., Chen B. (2011). Advances in the understanding of the structure and function of ER-α36,a novel variant of human estrogen receptor-α. J. Steroid Biochem. Mol. Biol..

[16] Cowley S.M., Hoare S., Mosselman S., Parker M.G. (1997). Estrogen receptors α and β form heterodimers on DNA. J. Biol. Chem..

[17] Tamrazi A., Carlson K.E., Daniels J.R., Hurth K.M., Katzenellenbogen J.A. (2002). Estrogen receptor dimerization: Ligand binding regulates dimer affinity and dimer dissociation rate. Mol. Endocrinol..

[18] Groner A.C., Brown M. (2017). Role of steroid receptor and coregulator mutations in hormone-dependent cancers. J. Clin. Investig..

[19] Szklarczyk D., Morris J.H., Cook H., Kuhn M., Wyder S., Simonovic M., Santos A., Doncheva N.T., Roth A., Bork P. (2017). The STRING database in 2017: Quality-controlled protein-protein association networks, made broadly accessible. Nucleic Acids Res..

[20] Cavailles V., Dauvois S., L’Horset F., Lopez G., Hoare S., Kushner P.J., Parker M.G. (1995). Nuclear factor RIP140 modulates transcriptional activation by the estrogen receptor. EMBO J..

[21] Lavinsky R.M., Jepsen K., Heinzel T., Torchia J., Mullen T.M., Schiff R., Del-Rio A.L., Ricote M., Ngo S., Gemsch J. (1998). Diverse signaling pathways modulate nuclear receptor recruitment of N-CoR and SMRT complexes. Proc. Natl. Acad. Sci. USA.

[22] Johansson L., Thomsen J.S., Damdimopoulos A.E., Spyrou G., Gustafsson J.A., Treuter E. (1999). The orphan nuclear receptor SHP inhibits agonist-dependent transcriptional activity of estrogen receptor α and β. J. Biol. Chem..

[23] Sun S., Yang X., Wang Y., Shen X. (2016). In Vivo Analysis of Protein–Protein Interactions with Bioluminescence Resonance Energy Transfer (BRET): Progress and Prospects. Int. J. Mol. Sci..

[24] Komatsu N., Terai K., Imanishi A., Kamioka Y., Sumiyama K., Jin T., Okada Y., Nagai T., Matsuda M.A. (2018). platform of BRET-FRET hybrid biosensors for optogenetics, chemical screening, and in vivo imaging. Sci. Rep..

[25] Michnick S.W., Ear P.H., Landry C., Malleshaiah M.K., Messier V. (2010). A toolkit of protein-fragment complementation assays for studying and dissecting large-scale and dynamic protein-protein interactions in living cells. Methods Enzymol..

[26] Yurlova L., Derks M., Buchfellner A., Hickson I., Janssen M., Morrison D., Stansfield I., Brown C.J., Ghadessy F.J., Lane D.P. (2014). The fluorescent two-hybrid assay to screen for protein-protein interaction inhibitors in live cells: Targeting the interaction of p53 with Mdm2 and Mdm4. J. Biomol. Screen..

[27] Malleshaiah M., Tchekanda E., Michnick S.W. (2016). Real-Time Protein-Fragment Complementation Assays for Studying Temporal, Spatial, and Spatiotemporal Dynamics of Protein-Protein Interactions in Living Cells. Cold Spring Harb. Protoc..

[28] Wang S., Ding M., Xue B., Hou Y., Sun Y. (2018). Live Cell Visualization of Multiple Protein-Protein Interactions with BiFC Rainbow. ACS Chem. Biol..

[29] Choi M., Baek J., Han S.B., Cho S. (2018). Facile Analysis of Protein-Protein Interactions in Living Cells by Enriched Visualization of the P-body. BMB Rep..

[30] Lin T., Scott B.L., Hoppe A.D., Chakravarty S. (2018). FRETting About the Affinity of Bimolecular Protein-Protein Interactions. Protein Sci..

[31] Masters S.C. (2004). Co-immunoprecipitation from transfected cells. Methods Mol. Biol..

[32] Tang Z., Takahashi Y. (2018). Analysis of Protein-Protein Interaction by Co-IP in Human Cells. Methods Mol. Biol..

[33] Mohammed H., D’Santos C., Serandour A.A., Ali H.R., Brown G.D., Atkins A., Rueda O.M., Holmes K.A., Theodorou V., Robinson J.L. (2013). Endogenous purification reveals GREB1 as a key estrogen receptor regulatory factor. Cell Rep..

[34] Boute N., Jockers R., Issad T. (2002). The use of resonance energy transfer in high-throughput screening: BRET versus FRET. Trends Pharmacol. Sci..

[35] Lohse M.J., Bünemann M., Hoffmann C., Vilardaga J.P., Nikolaev V.O. (2007). Monitoring receptor signaling by intramolecular FRET. Curr. Opin. Pharmacol..

[36] Förster T. (1946). Energy transport and fluorescence. Naturwissenschafien.

[37] Emmanouilidou E., Teschemacher A.G., Pouli A.E., Nicholls L.I., Seward E.P., Rutter G.A. (1999). Imaging Ca^2+^ concentration changes at the secretory vesicle surface with a recombinant targeted cameleon. Curr. Biol..

[38] Shinoda H., Shannon M., Nagai T. (2018). Fluorescent Proteins for Investigating Biological Events in Acidic Environments. Int. J. Mol. Sci..

[39] Xu Y., Piston D.W., Johnson C.H. (1999). A bioluminescence resonance energy transfer (BRET) system: Application to interacting circadian clock proteins. Proc. Natl. Acad. Sci. USA.

[40] Jensen A.A., Hansen J.L., Sheikh S.P., Bräuner-Osborne H. (2002). Probing intermolecular protein-protein interactions in the calcium-sensing receptor homodimer using bioluminescence resonance energy transfer (BRET). Eur. J. Biochem..

[41] Eidne K.A., Kroeger K.M., Hanyaloglu A.C. (2002). Applications of novel resonance energy transfer techniques to study dynamic hormone receptor interactions in living cells. Trends Endocrinol. Metab..

[42] De A., Jasani A., Arora R., Gambhir S.S. (2013). Evolution of BRET Biosensors from Live Cell to Tissue-Scale In Vivo Imaging. Front. Endocrinol. (Lausanne).

[43] Dragulescu-Andrasi A., Chan C.T., De A., Massoud T.F., Gambhir S.S. (2011). Bioluminescence resonance energy transfer (BRET) imaging of protein-protein interactions within deep tissues of living subjects. Proc. Natl. Acad. Sci. USA.

[44] Tung J.K., Berglund K., Gutekunst C.A., Hochgeschwender U., Gross R.E. (2016). Bioluminescence imaging in live cells and animals. Neurophotonics.

[45] Michelini E., Mirasoli M., Karp M., Virta M., Roda A. (2004). Development of a bioluminescence resonance energy-transfer assay for estrogen-like compound in vivo monitoring. Anal. Chem..

[46] Yasgar A., Jadhav A., Simeonov A., Coussens N.P. (2016). AlphaScreen-Based Assays: Ultra-High-Throughput Screening for Small-Molecule Inhibitors of Challenging Enzymes and Protein-Protein Interactions. Methods Mol. Biol..

[47] Rouleau N., Turcotte S., Mondou M.H., Roby P., Bossé R. (2003). Development of a versatile platform for nuclear receptor screening using AlphaScreen. J. Biomol. Screen..

[48] Yalow R.S., Berson S.A. (1960). Immunoassay of endogenous plasma insulin in man. Clin. Investig..

[49] Griffiths J., Rippe D.F., Panfili P.R. (1982). Comparison of enzymelinked immunosorhent assay and radioimmunoassay for prostatespecific acid phosphatase in prostatic disease. Clin. Chem..

[50] Holt J.A., Bolanos J. (1986). Enzyme-linked immunochemical measurement of estrogen receptor in gynecologic tumors, and an overview of steroid receptors in ovarian carcinoma. Clin. Chem..

[51] Porstmann T., Kiessig S.T. (1992). Enzyme immunoassay techniques. An overview. J. Immunol. Methods.

[52] Towbin H., Staehelin T., Gordon J. (1979). Electrophoretic transfer of proteins from polyacrylamide gels to nitrocellulose sheets: Procedure and some applications. Proc. Natl. Acad. Sci. USA.

[53] Burnette W.N. (1981). “Western Blotting”: Electrophoretic Transfer of Proteins from Sodium Dodecyl Sulfate-Polyacrylamide Gels to Unmodified Nitrocellulose and Radiographic Detection with Antibody and Radioiodinated Protein A. Anal. Biochem..

[54] Peluso P., Wilson D.S., Do D., Tran H., Venkatasubbaiah M., Quincy D., Heidecker B., Poindexter K., Tolani N., Phelan M. (2003). Optimizing antibody immobilization strategies for the construction of protein microarrays. Anal. Biochem..

[55] Watanabe M., Guo W., Zou S., Sugiyo S., Dubner R., Ren K. (2005). Antibody array analysis of peripheral and blood cytokine levels in rats after masseter inflammation. Neurosci. Lett..

[56] de Jager W., te Velthuis H., Prakken B.J., Kuis W., Rijkers G.T. (2003). Simultaneous detection of 15 human cytokines in a single sample of stimulated peripheral blood mononuclear cells. Clin. Diagn. Lab. Immunol..

[57] Coons A.H., Creech H.J., Jones R.N. (1941). Immunological properties of an antibody containing a fluorescent group. Proc. Soc. Exp. Biol. Med..

[58] Jacobs T.W., Gown A.M., Yaziji H., Barnes M.J., Schnitt S.J. (1999). Specificity of HercepTest in determining HER-2/neu status of breast cancers using the United States Food and Drug Administration-approved scoring system. J. Clin. Oncol..

[59] Taylor C.R. (2014). Predictive Biomarkers and Companion Diagnostics. The Future of Immunohistochemistry—‘In situ proteomics’, or just a ‘stain’?. Appl. Immunohistochem. Mol. Morphol..

[60] Erskine L.P., Mary L.M., Hyatt A.D., Eaton B.T. (1993). Chapter 1: Immune complexing. Immuno-Gold Electron Microscopy in Virus Diagnosis and Research.

[61] Bridoux F., Leung N., Hutchison C.A., Touchard G., Sethi S., Fermand J.P., Picken M.M., Herrera G.A., Kastritis E., Merlini G. (2015). Diagnosis of monoclonal gammopathy of renal significance. Kidney Int..

[62] Figueres M.L., Beaume J., Vuiblet V., Rabant M., Bassilios N., Herody M., Touchard G., Noël L.H. (2015). Crystalline light chain proximal tubulopathy with chronic renal failure and silicone gel breast implants: 1 case report. Hum. Pathol..

[63] Hendrix A., De Wever O. (2013). Rab27 GTPases Distribute Extracellular Nanomaps for Invasive Growth and Metastasis: Implications for Prognosis and Treatment. Int. J. Mol. Sci..

[64] Kocanova S., Mazaheri M., Caze-Subra S., Bystricky K. (2010). Ligands specify estrogen receptor α nuclear localization and degradation. BMC Cell Biol..

[65] Söderberg O., Gullberg M., Jarvius M., Ridderstrale K., Leuchowius K.J., Jarvius J., Wester K., Hydbring P., Bahram F., Larsson L.G. (2006). Direct observation of individual endogenous protein complexes in situ by proximity ligation. Nat. Methods.

[66] Söderberg O., Leuchowius K.J., Gullberg M., Jarvius M., Weibrecht I., Larsson L.G., Landegren U. (2008). Characterizing proteins and their interactions in cells and tissues using the in situ proximity ligation assay. Methods.

[67] Iwabuchi E., Miki Y., Ono K., Onodera Y., Suzuki T., Hirakawa H., Ishida T., Ohuchi N., Sasano H. (2017). In situ detection of estrogen receptor dimers in breast carcinoma cells in archival materials using proximity ligation assay (PLA). J. Steroid Biochem. Mol. Biol..

[68] Iwabuchi E., Miki Y., Ono K., Onodera Y., Sasano H. (2017). In Situ Evaluation of Estrogen Receptor Dimers in Breast Carcinoma Cells: Visualization of Protein-Protein Interactions. Acta Histochem. Cytochem..

[69] Ogawa S., Inoue S., Watanabe T., Orimo A., Hosoi T., Ouchi Y., Muramatsu M. (1998). Molecular cloning and characterization of human estrogen receptor βcx: A potential inhibitor ofestrogen action in human. Nucleic Acids Res..

[70] Omoto Y., Eguchi H., Yamamoto-Yamaguchi Y., Hayashi S. (2003). Estrogen receptor (ER) β1 and ERβcx/β2 inhibit ERα function differently in breast cancer cell line MCF7. Oncogene.

[71] Leung Y.K., Mak P., Hassan S., Ho S.M. (2006). Estrogen receptor (ER)-β isoforms: A key to understanding ER-β signaling. Proc. Natl. Acad. Sci. USA.

[72] Peng B., Lu B., Leygue E., Murphy L.C. (2003). Putative functional characteristics of human estrogen receptor-β isoforms. J. Mol. Endocrinol..

[73] Flanders K.C., Heger C.D., Conway C., Tang B., Sato M., Dengler S.L., Goldsmith P.K., Hewitt S.M., Wakefield L.M. (2014). Brightfield proximity ligation assay reveals both canonical and mixed transforming growth factor-β/bone morphogenetic protein Smad signaling complexes in tissue sections. J. Histochem. Cytochem..

[74] Zieba A., Wählby C., Hjelm F., Jordan L., Berg J., Landegren U., Pardali K. (2010). Bright-field microscopy visualization of proteins and protein complexes by in situ proximity ligation with peroxidase detection. Clin. Chem..

[75] Mehraein-Ghomi F., Kegel S.J., Church D.R., Schmidt J.S., Reuter Q.R., Saphner E.L., Basu H.S., Wilding G. (2014). Targeting androgen receptor and JunD interaction for prevention of prostate cancer progression. Prostate.

[76] Iwabuchi E., Miki Y., Kanai A., Miyashita M., Kijima G., Hirakawa H., Suzuki T., Ishida T., Sasano H. (2018). The interaction between carcinoembryonic antigen-related cell adhesion molecule 6 and HER2 is associated with therapeutic efficacy of trastuzumab in breast cancer. J. Pathol..

[77] Kanthala S., Banappagari S., Gokhale A., Liu Y.Y., Xin G., Zhao Y., Jois S. (2015). Novel Peptidomimetics for Inhibition of HER2:HER3 Heterodimerization I HER2-Positive Breast Cancer. Chem. Biol. Drug Des..

[78] Falkenberg N., Anastasov N., Höfig I., Bashkueva K., Lindner K., Höfler H., Rosemann M., Aubele M. (2015). Additive impact of HER2-/PTK6-RNAi on interactions with HER3 or IGF-1R leads to reduced breast cancer progression in vivo. Mol. Oncol..

[79] Barros F.F., Abdel-Fatah T.M., Moseley P., Nolan C.C., Durham A.C., Rakha E.A., Chan S., Ellis I.O., Green A.R. (2014). Characterisation of HER heterodimers in breast cancer using in situ proximity ligation assay. Breast Cancer Res. Treat..

[80] Spears M., Taylor K.J., Munro A.F., Cunningham C.A., Mallon E.A., Twelves C.J., Cameron D.A., Thomas J., Bartlett J.M. (2012). In situ detection of HER2:HER2 and HER2:HER3 protein-protein interactions demonstrates prognostic significance in early breast cancer. Breast Cancer Res. Treat..

[81] Ellebaek S., Brix S., Grandal M., Lantto J., Horak I.D., Kragh M., Poulsen T.T. (2016). Pan-HER-An antibody mixture targeting EGFR, HER2 and HER3 abrogates preformed and ligand-induced EGFR homo- and heterodimers. Int. J. Cancer.

[82] Bahjat M., Bloedjes T.A., van der Veen A., de Wilde G., Maas C., Guikema J.E.J. (2017). Detection and Visualization of DNA Damage-induced Protein Complexes in Suspension Cell Cultures Using the Proximity Ligation Assay. J. Vis. Exp..

[83] Fredriksson S., Horecka J., Brustugun O.T., Schlingemann J., Koong A.C., Tibshirani R., Davis R.W. (2008). Multiplexed proximity ligation assays to profile putative plasma biomarkers relevant to pancreatic and ovarian cancer. Clin. Chem..

[84] Leuchowius K.J., Weibrecht I., Landegren U., Gedda L., Soderberg O. (2009). Flow cytometric in situ proximity ligation analyses of proteininteractions and post-translational modification of the epidermal growth factor receptor family. Cytom. A.

[85] Blokzijl A., Friedman M., Pontén F., Landegren U. (2010). Profiling protein expression and interactions: Proximity ligation as a tool for personalized medicine. J. Intern. Med..

[86] Ward E.N., Pal R. (2017). Image scanning microscopy: An overview. J. Microsc..

[87] Vangindertael J., Camacho R., Sempels W., Mizuno H., Dedecker P., Janssen K.P.F. (2018). An introduction to optical super-resolution microscopy for the adventurous biologist. Methods Appl. Fluoresc..

[88] Htun H., Holth L.T., Walker D., Davie J.R., Hager G.L. (1999). Direct visualization of the human estrogen receptor α reveals a role for ligand in the nuclear distribution of the receptor. Mol. Biol. Cell.

[89] Zhao Y., Goto K., Saitoh M., Yanase T., Nomura M., Okabe T., Takayanagi R., Nawata H. (2002). Activation function-1 domain of androgen receptor contributes to the interaction between subnuclear splicing factor compartment and nuclear receptor compartment. Identification of the p102 U5 small nuclear ribonucleoprotein particle-binding protein as a coactivator for the receptor. J. Biol. Chem..

[90] Ochiai I., Matsuda K., Nishi M., Ozawa H., Kawata M. (2004). Imaging analysis of subcellular correlation of androgen receptor and estrogen receptor α in single living cells using green fluorescent protein color variants. Mol. Endocrinol..

[91] Sharp Z.D., Mancini M.G., Hinojos C.A., Dai F., Berno V., Szafran A.T., Smith K.P., Lele T.P., Ingber D.E., Mancini M.A. (2006). Estrogen-receptor-α exchange and chromatin dynamics are ligand- and domain-dependent. J. Cell Sci..

[92] Thorley J.A., Pike J., Rappoport J.Z., Cornea A., Conn P.M. (2014). Chapter 14: Super-resolution Microscopy: A Comparison of Commercially Available Options. Fluorescence Microscopy, Super-Resolution and Other Novel Techniques.

[93] Habuchi S. (2014). Super-resolution molecular and functional imaging of nanoscale architectures in life and materials science. Front. Bioeng. Biotechnol..

[94] Jonas K.C., Fanelli F., Huhtaniemi I.T., Hanyaloglu A.C. (2015). Single molecule analysis of functionally asymmetric G protein-coupled receptor (GPCR) oligomers reveals diverse spatial and structural assemblies. J. Biol. Chem..

[95] Jonas K.C., Chen S., Virta M., Mora J., Franks S., Huhtaniemi I., Hanyaloglu A.C. (2018). Temporal reprogramming of calcium signalling via crosstalk of gonadotrophin receptors that associate as functionally asymmetric heteromers. Sci. Rep..

[96] Kaufmann R., Müller P., Hildenbrand G., Hausmann M., Cremer C. (2011). Analysis of Her2/neu membrane protein clusters in different types of breast cancer cells using localization microscopy. J. Microsc..

[97] Hausmann M., Ilić N., Pilarczyk G., Lee J.H., Logeswaran A., Borroni A.P., Krufczik M., Theda F., Waltrich N., Bestvater F. (2017). Challenges for Super-Resolution Localization Microscopy and Biomolecular Fluorescent Nano-Probing in Cancer Research. Int. J. Mol. Sci..

[98] Borroto-Escuela D.O., Romero-Fernandez W., Garriga P., Ciruela F., Narvaez M., Tarakanov A.O., Palkovits M., Agnati L.F., Fuxe K. (2013). G protein-coupled receptor heterodimerization in the brain. Methods Enzymol..

[99] Jeanquartier F., Jean-Quartier C., Holzinger A. (2015). Integrated web visualization for protein-protein interaction databases. BMC Bioinform..

[100] Rodriguez A.L., Tamrazi A., Collins M.L., Katzenellenbogen J.A. (2004). Design, synthesis, and in vitro biological evaluation of small molecule inhibitors of estrogen receptor α coactivator binding. J. Med. Chem..

[101] Sun A., Moore T.W., Gunther J.R., Kim M.S., Rhoden E., Du Y., Fu H., Snyder J.P., Katzenellenbogen J.A. (2011). Discovering small-molecule estrogen receptor α/coactivator binding inhibitors: High-throughput screening, ligand development, and models for enhanced potency. ChemMedChem.

[102] Leclercq G., Gallo D., Cossy J., Laïos I., Larsimont D., Laurent G., Jacquot Y. (2011). Peptides targeting estrogen receptor α-potential applications for breast cancer treatment. Curr. Pharm. Des..

[103] Lempereur M., Majewska C., Brunquers A., Wongpramud S., Valet B., Janssens P., Dillemans M., Van Nedervelde L., Gallo D. (2016). Tetrahydro-iso-α Acids Antagonize Estrogen Receptor Alpha Activity in MCF-7 Breast Cancer Cells. Int. J. Endocrinol..

[104] Singh K., Munuganti R.S.N., Lallous N., Dalal K., Yoon J.S., Sharma A., Yamazaki T., Cherkasov A., Rennie P.S. (2018). Benzothiophenone Derivatives Targeting Mutant Forms of Estrogen Receptor-α in Hormone-Resistant Breast Cancers. Int. J. Mol. Sci..

[105] Thomas C., Gustafsson J.Å. (2015). Estrogen receptor mutations and functional consequences for breast cancer. Trends Endocrinol. Metab..

[106] Gimzewski J.K., Joachim C. (1999). Nanoscale science of single molecules using local probes. Science.

[107] Sitterberg J., Ozcetin A., Ehrhardt C., Bakowsky U. (2010). Utilising atomic force microscopy for the characterisation of nanoscale drug delivery systems. Eur. J. Pharm. Biopharm..

[108] Berthier A., Elie-Caille C., Lesniewska E., Delage-Mourroux R., Boireau W. (2011). Label-free sensing and atomic force spectroscopy for the characterization of protein-DNA and protein-protein interactions: Application to estrogen receptors. J. Mol. Recognit..

[109] Leclercq G., Laïos I., Elie-Caille C., Leiber D., Laurent G., Lesniewska E., Tanfin Z., Jacquot Y. (2017). ERα dimerization: A key factor for the weak estrogenic activity of an ERα modulator unable to compete with estradiol in binding assays. J. Recept. Signal Transduct. Res..

[110] Jacquot Y., Spaggiari D., Schappler J., Lesniewska E., Rudaz S., Leclercq G. (2017). ERE-dependent transcription and cell proliferation: Independency of these two processes mediated by the introduction of a sulfone function into the weak estrogen estrothiazine. Eur. J. Pharm. Sci..

